# Olanzapine-induced peripheral eosinophilia and eosinophilic pleural effusion: A case report

**DOI:** 10.1097/MD.0000000000009996

**Published:** 2018-02-16

**Authors:** Jing Huang, Yiming Yu, Wei Lin, Dandan Zhang, Zaichun Deng, Qunli Ding

**Affiliations:** a Department of Pharmacy, The Affiliated Hospital of Medical School of Ningbo University; b Department of Respiratory Medicine, The Affiliated Hospital of Medical School of Ningbo University, Ningbo, Zhejiang, China.

**Keywords:** adverse drug reaction, eosinophilic pleural effusion, EPE, olanzapine

## Abstract

**Rationale:**

Eosinophilic pleural effusion (EPE) is an eosinophil count ≥10% in pleural effusion, which is a rare condition in drug therapy.

**Patient concerns:**

We describe the case of a 70-year-old Alzheimer patient who was taking olanzapine for 2 months for the treatment of depression, and developed peripheral eosinophilia and bilateral EPE.

**Diagnoses:**

Olanzapine-induced peripheral eosinophilia and eosinophilic pleural effusion was diagnosed.

**Interventions:**

Olanzapine was discontinued, and repeated drainage of fluid from the pleural cavity was performed.

**Outcomes:**

All symptoms—as well as the EPE—were resolved 6 months later.

**Lessons:**

This case is a reminder that olanzapine may be a potential agent for EPE, and that this should be considered in clinical practice.

## 1 Introduction

Eosinophilic pleural effusion (EPE) is defined as an eosinophil count ≥ 10% in the pleural effusion. EPE occurs most commonly during conditions associated with the presence of blood or air in the pleural space, infections, inflammatory disorders, malignancy, and pulmonary embolism.^[1]^ However, EPE due to drug reaction is a less common occurrence. Certain drugs, including dantrolene, valproic acid, diltiazem, and simvastatin, are known to induce EPE.^[2]^ Our knowledge of drug-induced EPE comes almost exclusively from case reports. Publication of such cases can help identify drug-induced EPE. Here, we report a case of peripheral eosinophilia and bilateral EPE associated with olanzapine use in an Alzheimer patient.

## 2 Case report

**Figure 1:**
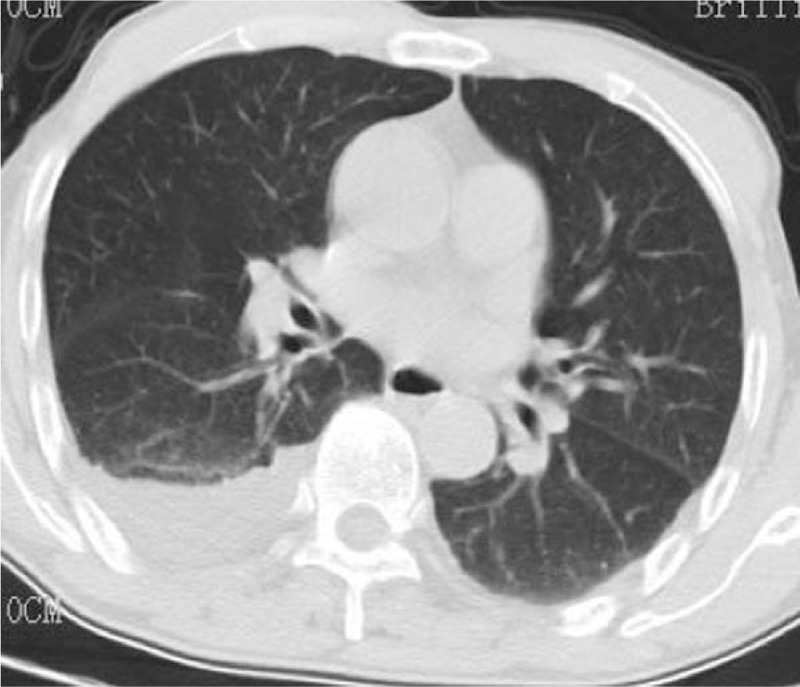
Chest computed tomography scan at presentation, showing a bilateral and massive left-side pleural effusion.

Two months prior to admission, a 70-year-old Chinese man was admitted to a rehabilitation unit and received medical treatment for the diagnosis of Alzheimer disease. His medications included memantine (20 mg daily PO), olanzapine (10 mg daily PO), paroxetine (20 mg daily PO), and piracetam (1.2 g 3 times daily PO). Two months later he presented with shortness of breath and chest pain. He was found to have a massive bilateral pleural effusion on the left side, which was detected by a chest computed tomography (CT) scan (Fig. 1), and was transferred to our respiratory department. On admission, the patient presented with no obvious dyspnea, cough, sputum, or other respiratory symptoms. He was fully conscious, and his vital signs were normal: temperature 36.8°C, pulse 96 bpm, respiratory rate 20 bpm, blood pressure 112/70 mm Hg, and blood oxygen saturation 98%. Pulmonary examination found dullness to percussion and diminished breath sounds on the right lower base with no pulmonary rales, confirmed as a pleural effusion by chest CT scan and B-scan ultrasonography. A chest CT scan showed no pleural thromboembolism. The remainder of the examination was unremarkable except for mild edema found in the lower limbs.

Laboratory tests showed a normal white cell count of 9500/μL (reference range 3500–10,000/μL) with significant eosinophilia of 1040/μL (10.9% of blood eosinophils), revealing a peripheral eosinophilia. Other laboratory test values showed a lactate deaminase level of 218 U/L (106–211 U/L), a C-reactive protein level of 11.9 mg/L (0–10 mg/L), and normal levels of hemoglobin and platelets. Liver function, renal function, blood coagulation function, serum rheumatologic work-up, tumor markers, antituberculosis antibody test, Schistosoma Japonium antibody test, and stool studies for parasite ova were all unremarkable/negative. No fever or rash was observed.

**Figure 2:**
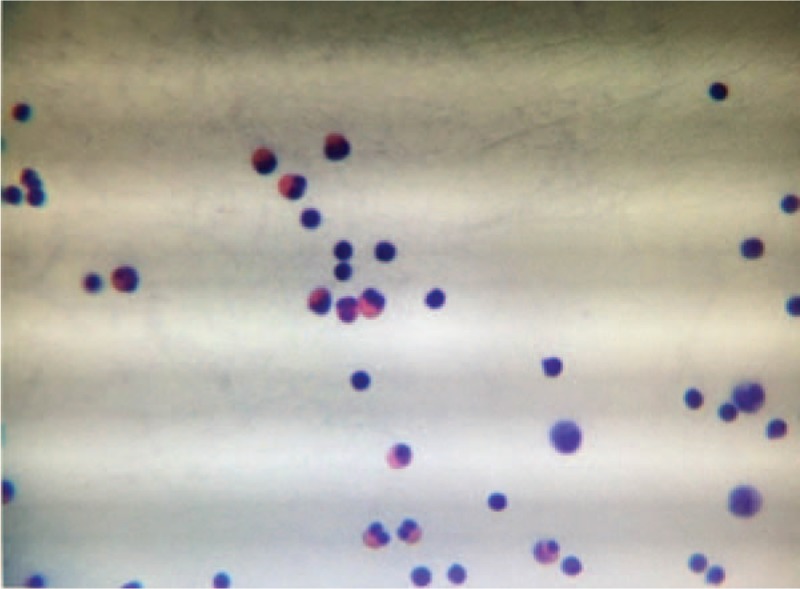
Thinprep cytologic test of pleural fluid, showing a significant eosinophilic infiltration.

A thoracentesis yielded an exudate serosanguinous pleural effusion that contained 6000/μL white blood cells with an excess of 43% of eosinophilia, 40,000/μL red blood cells, 454 U/L lactate deaminase (106–211 U/L), and 7.07 mmol/L glucose (2.5–4.5 mmol/L). A thinprep cytologic test of pleural fluid showed no evidence of malignant cells, but significant eosinophilic infiltration was observed (Fig. 2). Fluid bacterial cultures were negative. These examinations were repeated and similar results were obtained.

**Figure 3:**
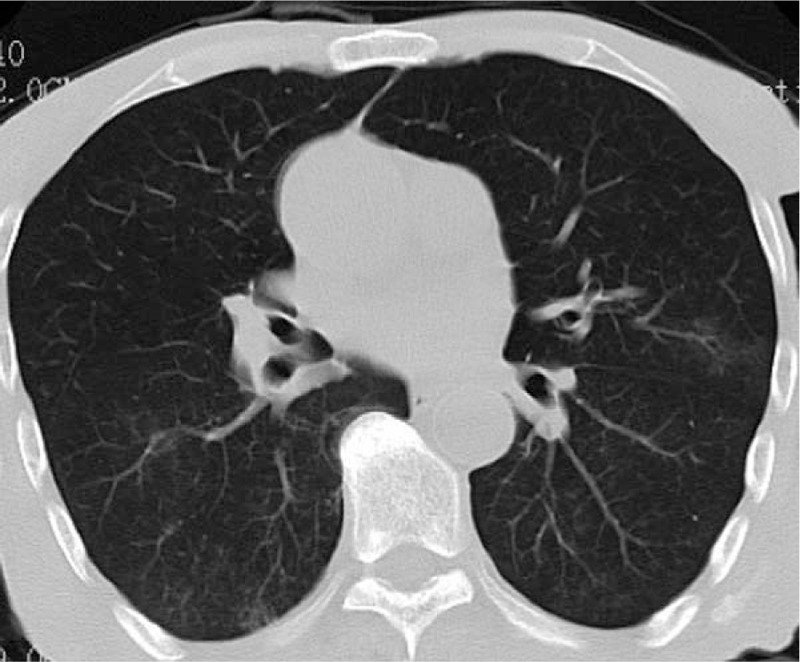
Chest computed tomography scan 6 months after olanzapine withdrawal, showing the resolution of the eosinophilic pleural effusion.

Examination results failed to yield any probable etiology of EPE, such as malignancy, infection, tuberculosis, pulmonary embolism, or autoimmune diseases. The suspicion of an iatrogenic pleural effusion due to drug reaction was considered given that the patient had taken several neurological agents for 2 months. Based on the above consideration, as well as suggestions from case reports in the literature, olanzapine was the only drug that was discontinued. This was combined with repeated drainage of the fluid from the pleural cavity (almost 1200 mL EPE was removed), resulting in a progressive improvement of our patient's condition and transference back to the rehabilitation unit. Six months later, reinspection results revealed that the peripheral eosinophilia and EPE had been resolved (Fig. 3).

## 3 Discussion

Idiopathic EPE due to drug reaction is rarely encountered in clinical practice. As a comprehensive review of drug-induced pleural effusion suggests, drugs such as valproic acid, propylthiouracil, isotretinoin, nitrofurantoin, bromocriptine, dantrolene, gliclazide, and mesalamine could be potential medications for EPE.^[2]^ To date, the list of the drugs implicated as potential causes of EPE has expanded, and (to the best of our knowledge) now also includes clozapine, olanzapine, tizanidine, trimipramine, fluoxetine, warfarin, vitamins B6/H, diltiazem, simvastatin, lisinopril, imidapril, daptomycin, and tosufloxacin.^[3–5]^ Most of these drugs are neurological agents, cardiovascular agents, or chemotherapeutic agents.

Olanzapine is an atypical antipsychotic medication approved by the US Food and Drug Administration (US FDA) and European Medicines Agency to treat schizophrenia and bipolar disorder. The most commonly reported adverse effects include weight gain, somnolence, dry mouth, dizziness, peripheral edema, orthostatic hypotension, tardive dyskinesia, seizures, and rash after sunlight exposure.^[6]^ However, olanzapine-induced EPE is rarely reported.

In the literature, we found 2 cases of olanzapine-associated EPE. Alagha et al^[7]^ reported a case of a 53-year-old male who had been treated with 10 mg olanzapine once daily for depression without other comorbidities for nearly 1 month. This patient began to experience a progressive and worsening left chest pain, as well as exertional dyspnea and dry cough, and had experienced these symptoms for 3 weeks before medical consultation. A left pleural effusion containing 75% eosinophils in the pleural cavity was detected, along with a small pericardial effusion. After withdrawal of olanzapine, the pleural effusion and other symptoms disappeared within 1 month. Another case report described an elderly patient with a history of depression with psychosis. This patient presented a right eosinophil-rich pleural effusion and peripheral eosinophilia after 12 months of olanzapine therapy. A diagnosis of olanzapine-induced EPE was suspected and 6 months later the pleural fluid resolved after olanzapine was discontinued.^[8]^ Finally, there was a case reporting a pericardial and bilateral pleural effusion associated with clozapine treatment in a patient who was also taking with olanzapine.^[9]^

In our case, olanzapine was used off-label for the treatment of depression in an Alzheimer patient, despite the fact that the US FDA has issued a black box warning about a higher mortality rate in elderly patients with dementia treated with olanzapine.^[6]^ The inference of olanzapine-induced EPE in our case was made with a causal relationship between medication intake and symptom onset, a lack of alternative explanations, and suggestions from case reports in the literature. This diagnosis was further supported by clinical improvement during the first several days after discontinuing olanzapine, as well as the complete resolution of EPE 6 months later. Utilizing the Naranjo Probability Scale,^[10]^ our patient had a score of 6, indicating that the EPE was “probably” caused by olanzapine (Table 1). Thus, our case may be the first case reported of EPE-induced by olanzapine in patient with Alzheimer disease.

**Table 1:**
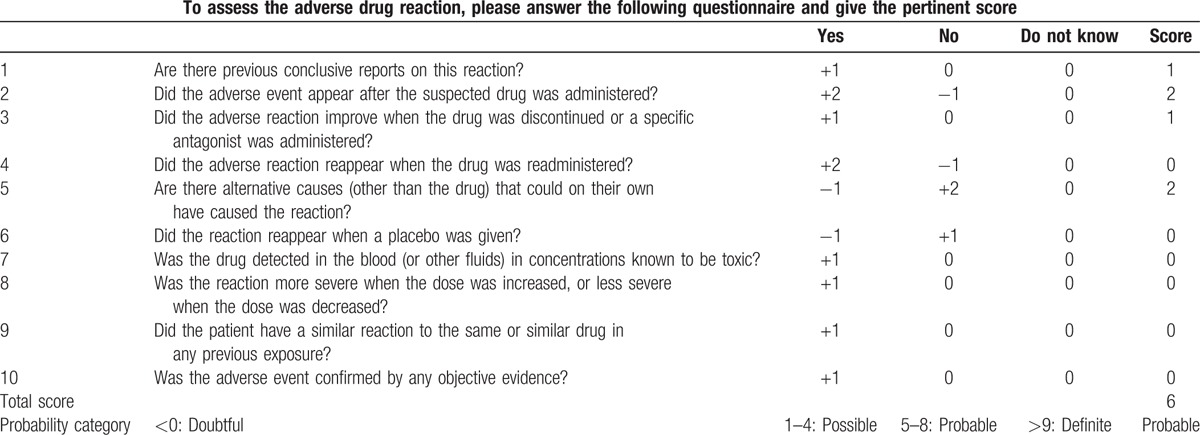
Naranjo Adverse Drug Reaction (ADR) Probability Scale.

The pathogenic mechanisms of drug-induced pleural effusions are to date not entirely understood. The proposed mechanisms include hypersensitivity or allergic reaction, direct toxic effect, increased oxygen free-radical production, suppression of antioxidant defense mechanism, and chemical-induced inflammation.^[2]^ The pathogenesis for olanzapine-induced EPE in our case remains speculative. An allergic reaction is strongly suspected because of the peripheral eosinophilia.

According to reports, the clinical symptoms of drug-induced EPE consist of chest pain, dyspnea, dry cough, and sometimes low-grade fever. The latency of EPE appearance can vary from hours to months, and potentially even years.^[11]^ In the 3 olanzapine-associated EPE cases, patients’ symptoms were seen varying from 1 month to 1 year of the initiation of olanzapine treatment and manifested as progressive breathlessness and chest pain. There was no-inclination of the lateral manifestation observed in these cases: 1 EPE was right-sided, 1 left-sided, and 1 bilateral with a mass in the left lobe. The complete resolution of the EPE takes 1 to 6 months.

In summary, the case we reported and those reviewed support that olanzapine may be a causative agent of EPE, and prompt discontinuation is suggested when it is strongly suspected.
